# Advancing Point-of-Care Diagnosis: Digitalizing Combinatorial Biomarker Signals for Lupus Nephritis

**DOI:** 10.3390/bios14030147

**Published:** 2024-03-18

**Authors:** Jiechang Guo, Aygun Teymur, Chenling Tang, Ramesh Saxena, Tianfu Wu

**Affiliations:** 1 Department of Biomedical Engineering, University of Houston, Houston, TX 77024, USA; jguo34@cougarnet.uh.edu (J.G.); arteymur@cougarnet.uh.edu (A.T.); ctang9@central.uh.edu (C.T.); 2 Department of Computer Science, University of Houston, Houston, TX 77024, USA; 3 Department of Internal Medicine, UT Southwestern Medical Center, Dallas, TX 75390, USA; ramesh.saxena@utsouthwestern.edu

**Keywords:** colorimetric microarray, smartphone application, clinical diagnostics, biomarker, image processing, point-of-care testing

## Abstract

To improve the efficiency and patient coverage of the current healthcare system, user-friendly novel homecare devices are urgently needed. In this work, we developed a smartphone-based analyzing and reporting system (SBARS) for biomarker detection in lupus nephritis (LN). This system offers a cost-effective alternative to traditional, expensive large equipment in signal detection and quantification. This innovative approach involves using a portable and affordable microscopic reader to capture biomarker signals. Through smartphone-based image processing techniques, the intensity of each biomarker signal is analyzed. This system exhibited comparable performance to a commercial Genepix scanner in the detection of two potential novel biomarkers of LN, VISG4 and TNFRSF1b. Importantly, this smartphone-based analyzing and reporting system allows for discriminating LN patients with active renal disease from healthy controls with the area-under-the-curve (AUC) value = 0.9 for TNFRSF1b and 1.0 for VSIG4, respectively, indicating high predictive accuracy.

## 1. Introduction

Lupus nephritis (LN), a severe manifestation of systemic lupus erythematosus (SLE), significantly compromises patient outcomes through immune-mediated renal damage, characterized by the deposition of immune complexes in the kidneys [1]. The progression of SLE to LN varies widely among patients, ranging in the degree of renal involvement, with 40% of these individuals eventually receiving a clinical diagnosis of LN [2]. The clinical presentations and laboratory parameters of patients with LN demonstrate significant variability, depending on disease activity. Active lupus nephritis (LNA) is characterized by ongoing renal inflammation and disease activity, often manifested clinically through proteinuria, hematuria, hypertension, and renal dysfunction, with significant urinary protein levels being a primary indicator of its active state [3]. The existing laboratory markers for LN, such as proteinuria, urine protein-to-creatinine ratio, creatinine clearance, anti-dsDNA, and complement levels, have challenges in accurately detecting renal activity and damage due to their limited specificity and sensitivity [4].

Given the complex interplay between the immune response and renal pathology in lupus nephritis (LN), the detection and management of this condition present significant clinical challenges. The immune system’s activity in SLE leads to the formation and deposition of immune complexes in the kidneys, triggering inflammation and damage that, without precise and early intervention, often progresses to LN with poor prognostic outcomes [1]. The variability in the progression and presentation of LN among patients further complicates its diagnosis and management, necessitating a dynamic and sensitive approach to detect changes in renal function and disease activity. This diagnostic challenge highlights a gap in the current diagnostic methodologies and underscores the need for more sensitive and specific biomarkers or testing approaches in the management of this condition. To effectively manage the progression of disease in these patients, the development of diagnostic approaches is crucial in detecting renal flares and consistent monitoring of disease progression.

Although invasive procedures like kidney biopsies remain the established gold standard for disease diagnosis, the associated risks and potential for serious complications underscore the need for safer alternatives [5]. Non-invasive methodologies, using serum, urine, or saliva samples, offer a promising solution by employing non-invasive techniques to detect novel biomarkers present in bodily fluids [6]. Identifying the changes in protein expression in biological fluids presents a viable avenue for early disease detection and could serve as an adjunctive method alongside histopathology.

Over the past decade, the field of proteomics has significantly advanced clinical research, enhancing our understanding of disease mechanisms, facilitating the discovery of novel biomarkers for early diagnosis, and improving the monitoring of disease progression. The combination of two-dimensional gel electrophoresis (2D-PAGE) and mass spectrometry (MS)-based protein identification has served as the leading approach for proteomic analysis [7]. Recently, the mass spectrometry method has been enhanced by the advent of protein microarrays, an emerging proteomic technology that allows for the detailed investigation of specific protein–protein interactions associated with distinct disease conditions [8]. Despite the precision of mass spectrometry in identifying specific proteins and peptides, its application encounters significant challenges, where the presence of highly abundant proteins can significantly impede the detection of proteins with lower abundance [9].

Conversely, affinity-based methodologies, utilizing high-affinity ligands such as antibodies, offer a solution to these challenges. By selectively binding to their target proteins, these ligands enable the precise identification and quantification of low-abundance proteins [10]. This specificity is achieved through the use of antibodies or antigens that have been designed to recognize and bind to specific protein markers, thereby overcoming the limitations posed by the abundance of other proteins in the sample. Moreover, while mass spectrometry presents cost-related and operational challenges, the development and utilization of biomarker panels are notably less expensive and user-friendly, offering a practical alternative for widespread diagnostic applications [11].

As our understanding of the determinants influencing physiological processes deepens, there emerges a need for the screening and validation of biomarkers prior to finalizing diagnostic decisions. Point-of-care (POC) systems represent a pivotal advancement in this context, offering utility both within clinical environments for patient testing and as integral components of home-based diagnostic systems. The recent research has seen the development of specialized protein arrays designed to quantify specific biomarkers indicative of LN [12,13,14]. By enabling the precise measurement of these markers, such arrays are instrumental in distinguishing between different stages of LN, thereby offering a more tailored approach to patient management and prognosis evaluation. The emerging smartphone-based platforms offer promising solutions by providing portable, cost-effective, and connected alternatives for patient diagnostics across various medical fields [15]; for example, smartphone-based point-of-care testing of glucose and cholesterol [16], smartphone-based diagnostic platforms for rapid detection of viruses [17,18], as well as a variety of commercial smartphone-based devices and applications for personalized healthcare monitoring and management like Apple Heath, Premom, SmartBP, etc. Several biomarker detection methods utilizing smartphones, such as those discussed in [19,20,21], focus on fluorescence-based microarrays, showcasing the potential and promise of advancing diagnostic capabilities through accessible and portable technology. Furthermore, colorimetric detection systems, as applied in our study, offer several advantages over fluorescence-based methods, especially in resource-limited settings [22]. Colorimetric detection offers a simpler, more cost-effective alternative to fluorescence methods without the need for specialized equipment and is unaffected by photobleaching, a common issue in fluorescence detection systems [23]. This makes it more suitable for low-resource environments and home-care settings where complex equipment may not be readily available.

In this study, we introduce our smartphone-based analyzing and reporting system (SBARS) developed for the precise detection and quantification of biomarker signals in colorimetric microarray images. Based on our literature research [24] and our recent findings [25], two promising protein biomarkers, v-set and immunoglobulin domain containing-4 (VSIG4) and TNF receptor superfamily member 1 (BTNFRSF1b), were selected for the development of the smartphone-based multiplexed biomarker detection platform for lupus nephritis (LN). The rationale behind the development of this smartphone-based detection platform is rooted in its capacity to facilitate the early detection and ongoing management of LN, a critical concern in the realm of autoimmune diseases.

The SBARS utilizes advanced image processing algorithms that have been optimized to measure the intensity of biomarker spots with high precision, ensuring that the results are in direct correlation with those obtained through conventional scanning devices typically used in laboratory settings. We determined the optimal thresholds for our application for key biomarkers, specifically VSIG4, crucial for the prompt and accurate diagnosis of LN. SBARS is found to distinguish between active LN patients and healthy individuals with a notable predictive accuracy, evidenced by AUC values of 0.9 for TNFRSF1b and 1.0 for VSIG4. This innovative system represents a significant advancement in home-based healthcare technologies, offering a solution that is not only adaptable and reliable but also cost-effective for diagnostics and disease monitoring.

## 2. Materials and Methods

### 2.1. Patients, Clinical Samples, and Reagents

All human-subject-related procedures were performed following the institutionally approved IRB protocol (the University of Houston, IRB #STUDY00001299), and all the clinical samples used in this study are existing samples from the sample bank at the University of Houston. Serum samples from 10 healthy subjects and 20 lupus nephritis patients were used for the protein microarray analysis. Specifically, 10 patients with active LN (LNA), defined as having a systemic lupus erythematosus disease activity index (SLEDAI) score of >4 and SLEDAI renal domain (rSLEDAI) score of >0. The renal component of the SLEDAI (rSLEDAI) aggregates the renal-related aspects of the SLEDAI, encompassing conditions such as hematuria (>5 red blood cells per high-power field), pyuria (>5 white blood cells per high-power field), proteinuria (>0.5 g/24 h), and the presence of urinary casts.

Two protein biomarkers were used in the array in this study. V-set and immunoglobulin domain containing-4 (VSIG4, catalog no. MAB46461-100) and TNF receptor superfamily member 1B (TNFRSF1b, catalog no. DY726) antibodies were purchased from R&D Systems (Minneapolis, MN, USA). BSA-Biotin was used as a positive control, while phosphate-buffered saline (PBS) was used as a negative control. Based on the prior research [25], we selected the epoxy-modified polymer slide (STRATEC Consumables GmbH, Birkenfeld, Germany) for the immobilization of the antibodies and their subsequent detection.

For the detection of these proteins, a cocktail mixture of biotinylated antibodies, anti-VSIG4 (catalog no. BAD4646), and anti-TNFRSF1b (catalog no. BAF726) was purchased from R&D Systems. Biotinylated anti-human IgG (catalog no. 109-065-170) was also utilized in the mixture and purchased from Jackson ImmunoResearch (West Grove, PA, USA). The serum samples were diluted with Super G blocking buffer (catalog no. 105101) from Grace Bio-Labs (Bend, OR, USA) at an optimized dilution ratio. Streptavidin-HRP solution was used followed by SeramunBlau solution (Seramun Diagnostica GmbH, Heidesee, Germany) for colorimetric detection. Standard curves were generated based on serially diluted protein standards to determine the protein concentrations in the patient serum.

### 2.2. Biomarker Panel Chip

The chips/slides were loaded onto a non-contact microarray printing robot (sciFLEXARRAYER S3; Scienion GmbH, Berlin, Germany), and capture antibodies for each biomarker were printed in triplicate (drop volume: 450 ± 20 pl) on the slide using a PDC90 needle (Scienion GmbH, Berlin, Germany) at 25 ^°^C and 60% humidity. After printing, the slides were dried in the printing chamber overnight and assembled with SecureSeal Hybridization Chambers (Grace Bio-Labs, Bend, OR, USA). Each slide contained 16 identical arrays separated by the chamber to prevent sample cross-contamination. The assay method used for this array was the same as previously described [26]. Briefly, once dried overnight, the slide was brought to room temperature 30 min before testing. All 16 chambers on the assembled glass slide were blocked with 40 μL of blocking buffer at room temperature for one hour. Afterward, the blocking buffer was decanted, and properly diluted standards (recombinant proteins for VSIG4 and TNFRSF1b, serially diluted in 7 wells plus a blank) or serum samples were added into pre-configured wells for a two-hour incubation, then washed and incubated with streptavidin-HRP. Following additional washes, the slide was incubated with the substrate, SeramunBlau, to detect colorimetric signals that correspond to the printed antibody.

### 2.3. Instruments and Software

The colorimetric microarray images were scanned by a customized portable microscopic reader (ioLight, Hampshire, UK). The microscopic reader comes with the ioLight App, which is free for download from the Apple or Google Play app store and enables users to view, save, and store scanned images (Version 1.5 (224)). The microscope’s built-in camera captured the images, utilizing the ioLight application. The photos can be securely archived in a private photo gallery, ensuring enhanced protection and confidentiality. The size of the scanned color image is 1480 × 972 pixels in the JPEG format. The smartphone application was developed using Android Studio Giraffe and installed on a Samsung Galaxy A13 with Android version 13. The OpenCV 4.8.0 Android version was utilized to perform the biomarker signal detection task automatically.

**Figure 1:**
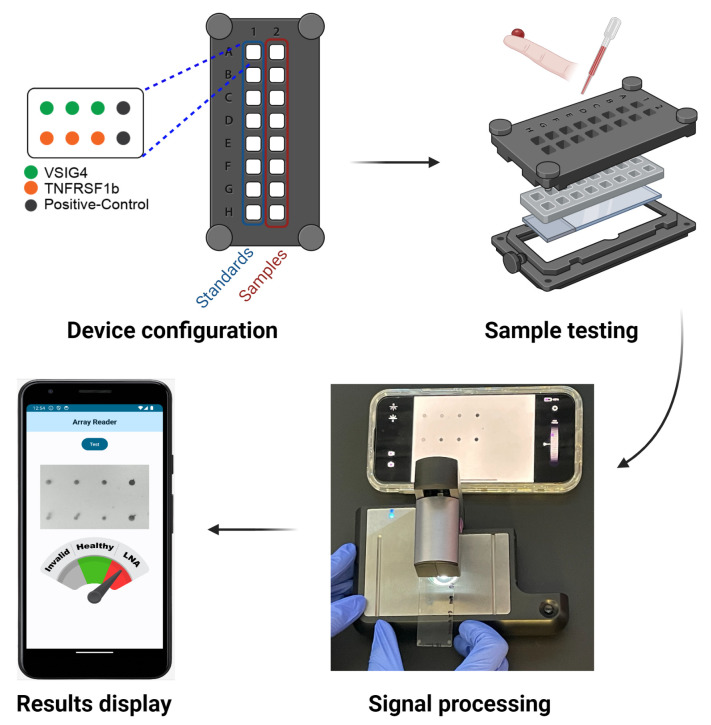
The pipeline of the smartphone-based quantification of biomarker panel signals for disease monitoring in lupus nephritis. The patient blood samples undergo microarray testing, where biomarkers are applied to slides and then imaged by the device. Standard curves are generated using serially diluted recombinant proteins and assayed side-by-side with the patient samples. The application processes the image, quantifying the biomarker signals for VSIG4 and TNFRSF1b. The result is displayed in a user-friendly interface, indicating the LN diagnosis status.

The overview pipeline of this paper is shown in Figure 1.

### 2.4. Biomarker Signal Detection

**Figure 2:**
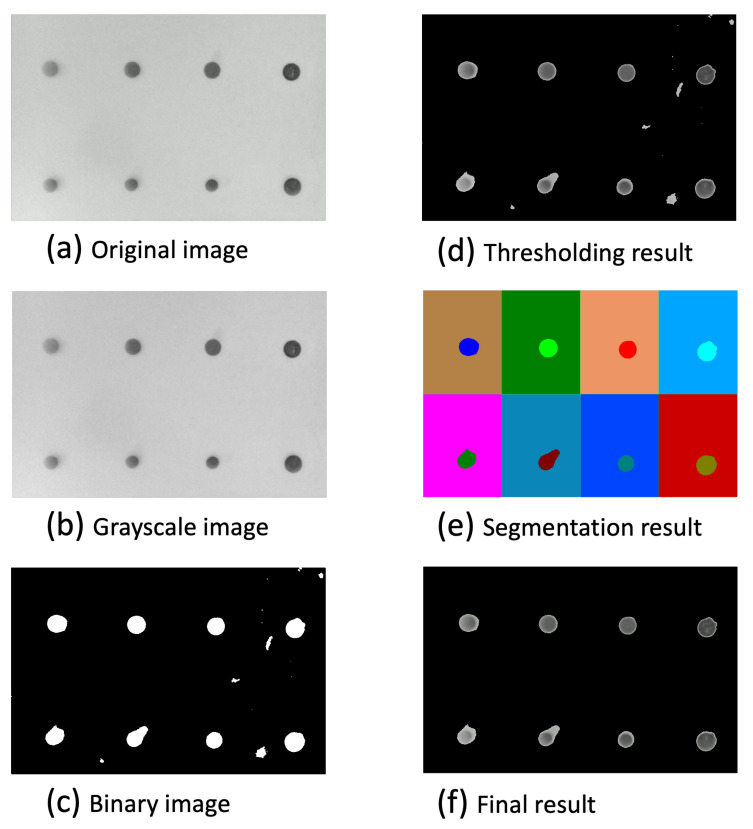
Sequence of the image processing for biomarker signal detection. (**a**) The original image scans. (**b**) The grayscale image. (**c**) The binary images using adaptive thresholding. (**d**) The thresholding resulted in the grayscale image without the background. (**e**) The image segmentation result using the connected component labeling technique. (**f**) The final result grayscale image without background.

Multiple advanced image processing methodologies were employed to enhance the accuracy and efficacy of biomarker signal detection in our study. The pipeline of the image processing for biomarker signal detection is shown in Figure 2. The colorimetric microarray image was first converted to the 8-bit single-channel grayscale image. Then, the grayscale image was blurred using a (21 × 21) Gaussian filter for better thresholding results that convolved the source image with the specified Gaussian Kernel. Instead of using global thresholding techniques like OSTU [27], the adaptive thresholding technique [28] was utilized, which calculates the threshold for each pixel based on its surrounding region, thereby offering enhanced performance for images with varying illumination conditions. Moreover, the quality of the resulting binary image is insufficient for direct pixel value extraction due to the presence of noise. The binary image is further divided into 2 × 4 small areas to be further processed. The connected component labeling and analysis technique was implemented to identify the contiguous areas within the image. Components presenting an area below a predefined threshold were classified as noise and subsequently filtered out, ensuring a more accurate signal detection. Finally, the intensity for each biomarker was calculated based on the mean of the pixel intensities of the labeled components.

### 2.5. Quantification of Biomarkers

**Figure 3:**
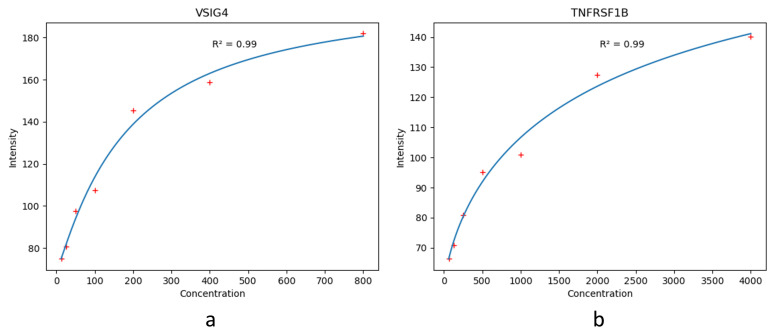
The fitted standard curves of the biomarkers. (**a**) The standard curve of VSIG4. (**b**) The standard curve of TNFRSF1b.

The intensity of each biomarker was quantitatively transformed into concentration values through the application of a fitted four-parameter logistic (4PL) curve model. It has four parameters that need to be estimated. This approach provides a more precise and robust estimation of the biomarker concentrations based on their respective intensity measurements. The equation for the model is

(1) $$y=d+\frac{a-d}{1+{\frac{x}{c}}^{b}}$$

where *x* is the intensity, and *y* is the concentration. The four estimated parameters are the following: *a* represents the obtained minimum value, *b* represents the obtained maximum value, *c* represents the points of inflection, and *d* represents the curve’s Hill’s slope. The fitted 4PL curve of each biomarker is shown in Figure 3.

Given the intensity of a sample biomarker, we use the rearranged Equation (1) to solve for concentration:

(2) $$x=c{\left(\frac{a-d}{y-d}-1\right)}^{\frac{1}{b}}$$

Based on the analysis of the distribution and performance of each biomarker discussed in the Results section, we selected the concentration of VSIG4 to classify the samples into two distinct groups: LN Active (LNA) patients and healthy individuals. This decision was guided by the differential performance observed in our analysis, where VSIG4 was identified as the most relevant and effective biomarker for this classification task in accurately distinguishing between the two groups. While TNFRSF1b was initially considered, its role in this specific context was found to be less pronounced, leading us to concentrate on the more indicative biomarker VSIG4 for LN disease monitoring.

Given the need to accurately identify LN patients, we use the following condition to classify samples as either *healthy* or *LNA* patients, where VSIG4_conc represents the concentration of VSIG4, and VSIG4_thre represents the optimal threshold of VSIG4:

$$\left\{\begin{array}{cc} Healthy & \mathrm{if}\;VSIG4\_conc\leq VSIG4\_thre\\ LNAPatient & otherwise \end{array}\right.$$

The optimal threshold VSIG4_thre was determined at 98.88, obtained through optimization to maximize sensitivity to ensure the system’s capability to accurately and reliably identify individuals with LNA.

### 2.6. Android Application

The disease-monitoring Android application was developed using Kotlin (223-1.8.0) and Jetpack Compose (1.4.0) in the free software Android Studio (Giraffe, 2022.3.1) powered by Google, Mountain View, CA, USA and JetBrains, Prague 4, Prague, Czechia. The OpenCV Android version was used to calculate the intensity for each biomarker.

The Android application provides an intuitive and simple user interaction to process a microarray sample image scanned from the microscopic reader. The application allows users to obtain the disease prediction result by clicking the “Test” button and then selecting the sample image obtained from the microscopic reader stored in the phone’s gallery. The application initially extracts the intensity data for each biomarker from the image. This intensity information is converted into concentration metrics utilizing an embedded, trained standard curve function. Subsequently, the concentration of the two biomarkers is used to predict if the sample is active, inactive, or healthy. The result includes the concentration for each biomarker shown in the table and the prediction result shown in the radial scale. If the sample is categorized as LN Active, the radial scale pointer is directed toward the “LNA” section highlighted in red. In the case of a healthy sample, the pointer aligns with the “Healthy” section in green. If the chosen image or sample is invalid, lacks a positive control signal, or has a mismatched image size with the microarray, the pointer points to the invalid section in gray.

## 3. Results

### 3.1. Image Processing Analysis

**Figure 4:**
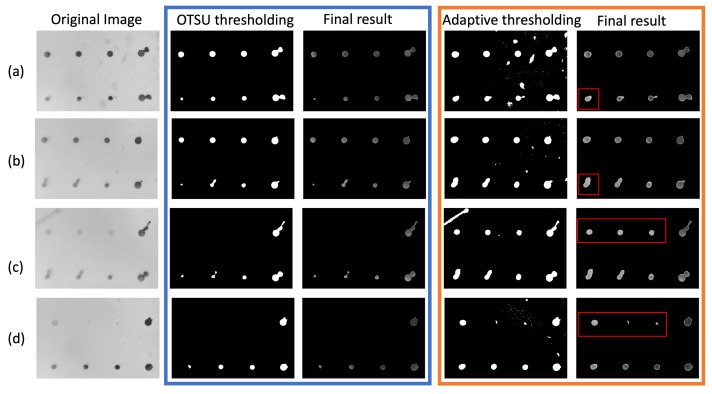
Comparison of OTSU thresholding and adaptive thresholding. The comparison includes four samples (**a**–**d**) showcasing the original image, the binary image result of OTSU thresholding, the final segmentation result after applying OTSU thresholding, the binary image result of adaptive thresholding, and the final segmentation result after applying adaptive thresholding. Samples (**a**,**b**) demonstrate instances where OTSU fails to fully capture biomarker spots, while samples (**c**,**d**) depict cases where OTSU misses subtle biomarker spots. The biomarker spots successfully captured by adaptive thresholding are highlighted using red rectangles.

Binary thresholding plays a vital role in biomarker intensity detection as it significantly influences the quality of differentiation between biomarker spots and background. The thresholding methods can be further divided into global thresholding techniques, like OTSU, and local thresholding. Although OTSU provides promising results for thresholding images, it may not be an ideal technique for a microarray image that contains different intensities throughout the image. Thus, in this work, the local thresholding technique, specifically adaptive thresholding, is employed to perform the binary thresholding task. Figure 4 shows the comparison between OSTU and adaptive thresholding. The experiments were by carried out by using the resulting binary image from OTSU thresholding and adaptive thresholding as input to perform the segmentation process. In Figure 4, the original image, binary images after OTSU thresholding, final segmentation results using OTSU thresholding, binary image after adaptive thresholding, and final segmentation results using adaptive thresholding are presented in sequence.

OTSU thresholding yields a clearer thresholding outcome compared to adaptive thresholding. However, it struggles to capture the biomarker spots with low intensity. For instance, as depicted in Figure 4a,b, the biomarker spots of TNFRSF1b are not adequately captured by the OTSU algorithm but exhibit well-defined boundaries with adaptive thresholding. In some extreme cases, such as those shown in Figure 4c,d, the OTSU technique failed to detect the VSIG4 biomarker spots with relatively low intensity, whereas adaptive thresholding successfully detects them. Following adaptive thresholding, the presence of some small dots may be observed, but these small dots were considered noise artifacts and eliminated after the segmentation step using the connected component detection algorithm.

### 3.2. Signal Stability and Correlation with Scanner Data

**Figure 5:**
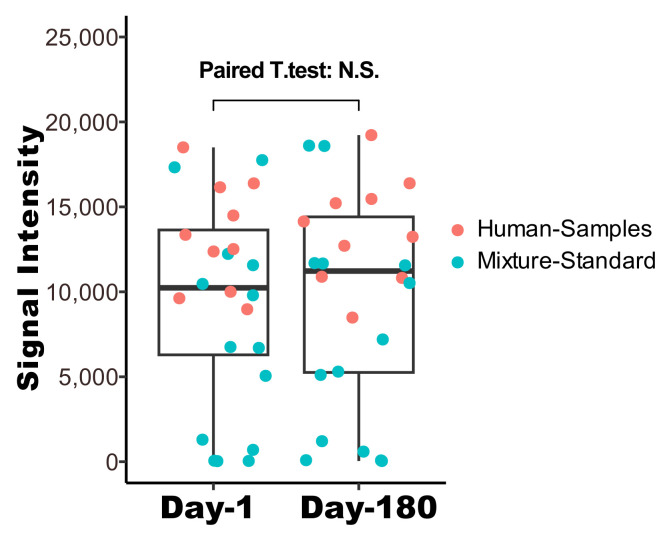
Long-term stability of biomarker panel array signals from colorimetric detection. The array images were captured on Day 1 and Day 180, respectively, and the signals were first quantified and then compared using a paired *t*-test. N.S.: statistically not significant.

The colorimetric images were additionally obtained six months after the initial experiment, with microarray slides stored at 4 degrees in a dark environment. We demonstrate in Figure 5 a comparative analysis of the signals collected at two distinct time points: Day 1 versus Day 180. A paired *t*-test was performed to compare the human samples and standard mixtures 180 days apart, and the results showed statistically no significant difference between the two groups, with *p*-value 0.13. These results illustrate an insignificant variation in signal intensity between the initial and subsequent measurements, thereby underscoring the robustness of the microarray signals for long-term preservation and the stability of the signals.

**Figure 6:**
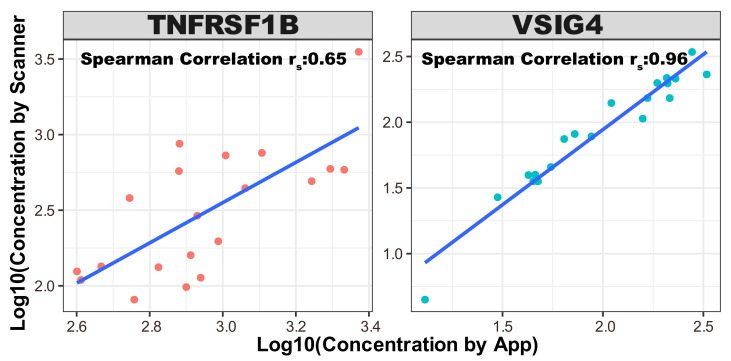
The Spearman’s correlation between scanner concentration and app intensity concentration. The Spearman’s rank correlation between app value and scanner value is 0.85. The Spearman’s rank correlation for individual biomarkers is 0.65 for TNFRSF1b and 0.94 for VSIG4.

The colorimetric intensity values, corresponding to each biomarker within the microarray images, were quantified utilizing a laboratory scanner software GenePix Pro 7, a widely utilized tool for scanning protein microarrays, and our proposed method, respectively. In order to investigate the potential statistical correlations between the scanner-derived data and the data obtained from the application, we computed the nonparametric Spearman’s rank correlation coefficient [29]. The correlation plot for each biomarker is shown in Figure 6. The Spearman’s rank correlation between app value and scanner value is 0.85. The Spearman’s rank correlation for individual biomarkers is 0.65 for TNFRSF1B and 0.94 for VSIG4.

The scatter plots highlight the alignment of concentration values across a logarithmic scale, with the linear fit indicating the degree to which the app-based method correlates with the scanner data. For VSIG4, the points cluster tightly along the line of best fit, whereas for TNFRSF1B, there is greater dispersion, reflecting the variance in correlation strength. The relatively high correlation observed for each biomarker suggests the potential feasibility of employing the app-based method as a cost-effective alternative to the conventional scanner for disease monitoring. The app’s ability to closely replicate the results of conventional scanners is particularly significant in contexts where access to high-end laboratory equipment may be limited.

### 3.3. Disease Monitoring of Patients

**Figure 7:**
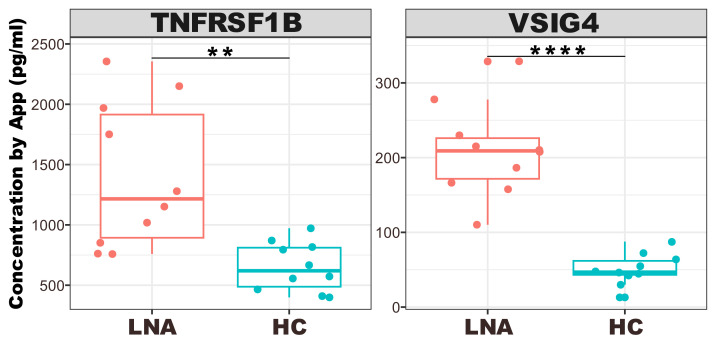
The box plot for concentration and two groups of data for each biomarker. LNA stands for LN active, and HC stands for healthy control. Asterisks designate the level of statistical significance: ** *p* < 0.01; **** *p* < 0.0001.

The box plots depicted in Figure 7 outline the quantified concentrations of TNFRSF1B and VSIG4 across two distinct groups: LN active (LNA) and healthy controls (HC).

Each box plot serves as a visual summary, capturing the central tendency, spread, and presence of outliers within each group. Specifically, for the VSIG4 biomarker, there is a discernible trend indicating the feasibility of differentiating LNA from HC, so it was identified as the most distinct and effective biomarker for LNA disease classification. This consistent elevation in VSIG4 levels among LNA patients compared to the HC group, along with a high degree of statistical significance, reinforces the discriminative power of this biomarker in reflecting disease activity. In the TNFRSF1B plot, there is a visible spread of concentrations among the LNA group, which suggests a variation in the expression levels of this biomarker in the disease state compared to the tight clustering seen in the HC group.

**Figure 8:**
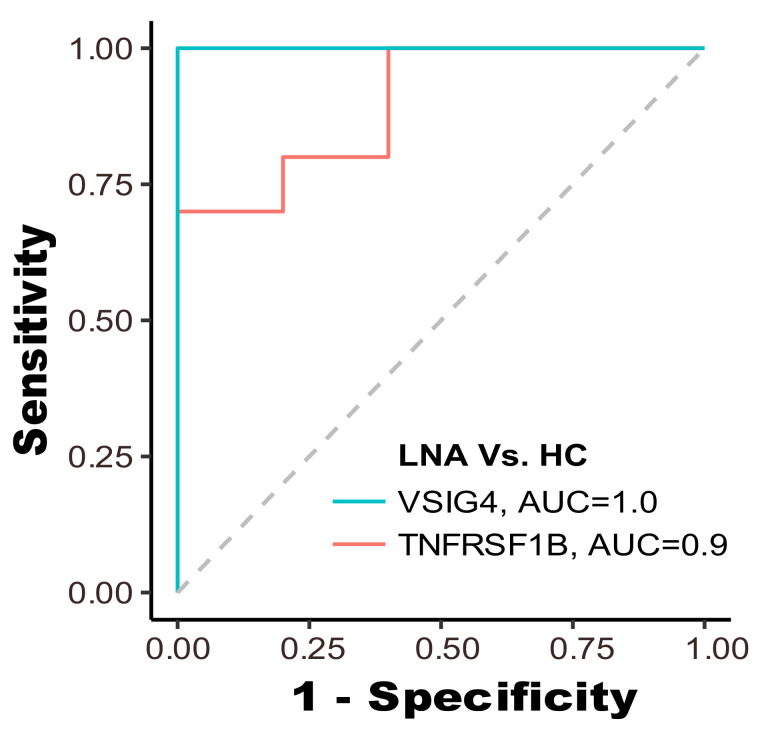
The ROC curves with AUC for individual biomarkers when performing the task of classifying LN active(LNA) and healthy control patients.

We further employed each biomarker as a feature for classifying samples into two groups: LN patients and healthy controls. The receiver operating characteristic (ROC) curve for each biomarker is displayed in Figure 8. The ROC curve for VSIG4, shown in blue, has an area-under-the-curve (AUC) of 1.0, indicating perfect discriminative ability, meaning it correctly identified all LNA cases and HC without any overlap. On the other hand, the TNFRSF1B curve, in red, with an AUC of 0.9, also shows high discriminative power, albeit with some overlap between the two groups. Both elevated AUC values signify a robust capability to accurately discern LN patients from those in the HC group.

## 4. Discussion

SLE is a complex autoimmune disease influenced by various factors, including genetics, environmental triggers, medications, hormonal changes, and gut microbiota composition [30]. As a result, no single biomarker can capture the diverse molecular and cellular pathways involved in the pathogenesis of LN. LN is associated with a wide range of clinical manifestations, including autoantibody production, proinflammatory cytokine release, organ damage (especially renal), immune cell subset activation changes, fatigue, fever, and proteinuria [31]. These diverse manifestations reflect the complex interplay of immune dysregulation and tissue damage in LN, making it challenging to assess disease activity and predict flares using a single biomarker.

A biomarker panel composed of multiple biomarkers reflecting different aspects of LN pathophysiology can enhance diagnostic accuracy compared to individual biomarkers alone. By integrating information from multiple pathways, such as immune activation, tissue damage, and inflammation, a biomarker panel can provide a more comprehensive assessment of disease activity and severity. Biomarker panels can also improve the prediction of disease progression and flares in LN by capturing subtle changes in disease activity over time. By monitoring dynamic changes in multiple biomarkers, clinicians can identify patients at increased risk of flare and intervene early to prevent disease exacerbation and organ damage. Advances in omics technologies, such as genomics, transcriptomics, proteomics, and metabolomics, have enabled the identification of novel biomarkers and the development of sophisticated biomarker panels for LN [32]. Machine learning algorithms can integrate the data from multiple omics platforms to generate highly accurate predictive models that account for the complex interactions between different molecular pathways in LN pathogenesis. In summary, a biomarker panel composed of multiple biomarkers reflecting various aspects of LN pathophysiology offers several advantages over individual biomarkers, including improved diagnostic accuracy, enhanced predictive value, and better monitoring of disease activity and progression. Omics research and machine learning modeling hold promise for the development of highly accurate and clinically relevant biomarker panels for LN diagnosis, monitoring, and prediction of flares.

Protein microarrays are increasingly utilized for detecting disease-specific autoantibodies or antigens, with significant implications for diagnostics and monitoring [33]. In this study, we examined 20 samples from LN patients and a healthy control group. Employing an image processing method, we quantified the intensity of each biomarker spot and compared it with the results from a conventional scanner. The results of our image processing analysis underscore the critical role of binary thresholding in biomarker intensity detection. Our findings revealed that while global thresholding techniques like OTSU are generally effective, they fall short in microarray images with varied intensities. To address these challenges, we implemented adaptive thresholding along with segmentation, which has shown superior performance in accurately separating biomarker spots from the background compared to traditional global thresholding methods [34]. This was particularly evident in the detection of low-intensity spots. The proposed image processing method for detecting colorimetric images can be adapted to fluorescence images with proper adjustment when converting the original image to a grayscale image. However, the colorimetric signal is more suitable for point-of-care situations with a portable microscopic reader because the traditional fluorescent system requires additional laser excitation components to capture signals.

Furthermore, the designed app incorporates a user-friendly interface that allows for easy navigation and operation, making it accessible to all users. This is crucial for widespread adoption, especially in resource-limited settings where sophisticated laboratory equipment may not be available [35,36]. The app also includes features for image capture, processing, and data analysis, all integrated seamlessly to provide a comprehensive solution for biomarker analysis. The robustness of our app-based method is further exemplified by its performance in preserving the integrity of biomarker signals over extended periods. Colorimetric images were additionally obtained six months after the initial experiment. The analysis revealed that a significant majority of the signals remained well-preserved, demonstrating minimal degradation over the six-month timeframe. The preservation of signal integrity over such an extended period is indicative of the robustness of the colorimetric method employed. This finding is particularly relevant in the context of longitudinal studies, where the ability to obtain reliable and consistent measurements over time can significantly enhance the understanding of chronic diseases. This aspect of the methodology is critical for ensuring that the app can be used reliably for long-term disease monitoring and study. This combination not only ensures high accuracy and reliability in biomarker intensity quantification but also makes the technology accessible and practical for widespread use.

Employing noninvasive methodologies, particularly serum-based assays, necessitates addressing the inherent challenges posed by serum constituents such as proteins, lipids, and electrolytes [37]. The presence of such interferences poses a challenge to biomarker detection as they can lead to non-specific binding or obscure the signals of the low-abundance biomarkers crucial for LN diagnosis. To mitigate these potential confounding effects, our approach involved the optimization of sample dilution protocols alongside the use of positive and negative control spots to ensure the specificity of our assay. Negative control spots (PBS) confirmed the absence of non-specific binding, while positive control spots (BSA-biotin) validated the assay’s ability to detect the intended targets. In the event of a testing failure, such as non-specific binding or failure to detect the biomarker signal accurately, the application is programmed to present an ‘Invalid’ result. This feature is a critical safeguard designed to ensure that each assay’s validity is maintained, with positive and negative controls integral to this verification process. The inclusion of these controls is essential for discerning true biomarker signals from potential background noise or assay anomalies, thereby enhancing the reliability of the test outcomes and preserving the integrity of the diagnostic process. Additionally, immunoaffinity-based depletion methods can be applied to the serum prior to testing, further refining the sample by selectively removing abundant proteins and enhancing the detection sensitivity for target biomarkers [38].

In terms of correlation with conventional scanner data, our application demonstrated a Spearman’s rank correlation of 0.85 overall. This indicates a strong correlation between our app-based method and the conventional scanner, underscoring the potential of our approach as a viable alternative for disease monitoring. Notably, individual biomarkers showed varying degrees of correlation, with TNFRSF1B at 0.65 and VSIG4 at a notable 0.94. The data points for TNFRSF1B display greater scatter when compared to VSIG4, indicating variability in the correlation. This dispersion could arise from several factors inherent to the nature of the TNFRSF1B biomarker, the quality of the antibodies used, the sample preparation, or the inherent variability in the serum samples tested. The variance in correlation strength for TNFRSF1B suggests that certain samples may have characteristics, such as variations in protein structure or concentration, that affect how well the biomarker binds to the antibody, leading to less consistent detection. Additionally, the performance of TNFRSF1B itself may be influenced by the dynamic range of the biomarker within the samples, potential cross-reactivity, or differing affinities of the antibodies used in the microarray construction, which can impact the robustness and reproducibility of the assay. The differential correlation strengths underscore the importance of selecting appropriate biomarkers for disease monitoring and diagnosis. Robust biomarkers like VSIG4, with high correlation strengths across different measurement platforms, are likely to be more reliable for clinical applications. In contrast, biomarkers like TNFRSF1B, which show more variability, may require additional consideration in assay development and data interpretation to ensure diagnostic accuracy.

In the selection of biomarkers integrated in our application, the rationale for focusing on VSIG4, while initially considering TNFRSF1b, stemmed from their complementary diagnostic properties. However, through rigorous testing and evaluation, we discovered inherent differences in their diagnostic efficacy when applied within the constraints of our mobile application platform that led to its exclusion. The decision to prioritize VSIG4 over TNFRSF1b emerged primarily due to the distinctive diagnostic attributes each biomarker presented. VSIG4’s high sensitivity was ideal for detecting active LN, a key factor for the intended application of our SBARS in early disease detection and management. This sensitivity is crucial for a condition like LN, where early detection can significantly influence treatment opinions and outcomes. On the other hand, TNFRSF1b, despite its initial promise, presented substantial challenges in achieving consistent and accurate detection within the mobile application framework. Upon examination of the scanner data, VSIG4 outperforms TNFRSF1B as a biomarker in this context for two primary reasons. First, VSIG4 exhibits a relatively higher median intensity value (median = 11,955.58) compared to TNFRSF1B (median = 8572.74). This difference is not trivial, as it directly impacts the reliability and sensitivity of the detection algorithm, which is designed to operate efficiently within a specific intensity range. Second, VSIG4 displays a wider distribution with a standard deviation of 5252.25, indicating a more balanced spread across the measured intensity range, in contrast to TNFRSF1B with a standard deviation of 2725.15. This broader distribution suggests that VSIG4’s signal variability, while higher, remains within an acceptable range for accurate detection by our smartphone-based system.

The compatibility of VSIG4 with the technical specifications of our point-of-care system is further underscored by the scanner data resolution and the image processing algorithm’s capacity. Given that the scale of measuring the signals is different in the two devices (0∼65,000 arbitrary units for the desk-top scanner and 0∼255 arbitrary units for the portable reader), the ability of the reader to capture lower signals may not be comparable to that of the scanner. VSIG4’s signal characteristics render it more compatible with the specifications of this POC system.

Then, with closer inspection of the original microarray image itself, it was observed that TNFRSF1b often appeared faded, a phenomenon attributed to its location at the lower end of the slides. Future studies could explore different image processing methods or adjustments in microarray design to improve its detection [21,39]. The issue with TNFRSF1b highlights an area for future methodological improvement, particularly in ensuring uniform sensitivity across different regions of the microarray slides. This exploration is crucial for enhancing the overall efficacy of microarray analyses in clinical diagnostics.

Moreover, the disease monitoring capabilities of our method revealed a clear distinction in VSIG4 levels between LN active and healthy controls, establishing its efficacy in distinguishing between these groups. The high AUC value of 1.0 for VSIG4 and 0.9 for TNFRSF1b further solidifies their potential as reliable biomarkers for LN classification. Such a high AUC value for VSIG4 suggests that this biomarker has excellent sensitivity and specificity for identifying lupus nephritis; nonetheless, the exceptional AUC value of 1.0 for VSIG4 warrants further investigation to confirm its validity in clinical settings. This high AUC value is likely due to the small cohort size utilized in this study. VSIG4’s diagnostic utility in lupus has been rigorously evaluated with a larger cohort in a previous study [25]. To further validate these findings, our portable smartphone-based diagnostic system will be subjected to a larger cohort in subsequent investigations to enhance the robustness of our results and conclusions.

## 5. Conclusions

In conclusion, our study not only demonstrates the efficacy of an app-based method for biomarker intensity quantification but also opens up new possibilities for its application in clinical diagnostics and monitoring. Our findings suggest that such mobile-based approaches could significantly contribute to personalized healthcare, particularly in facilitating home-based monitoring systems. Future research will focus on expanding the sample size, exploring a wider range of biomarkers, and integrating advanced data analysis techniques to further validate and enhance our method’s application in home-care based systems. This progression is aimed at establishing a robust home-care system that can adapt to the dynamic needs of patients and healthcare providers alike, facilitating early detection and ongoing management of various health conditions. 

## Data Availability

The data are contained within the article.

## Abbreviations

The following abbreviations are used in this manuscript:

LN	Lupus nephritis
SLE	Systemic lupus erythematosus
4PL	Four-parameter logistic
HC	Health control
LNA	Lupus nephritis active
ROC	Receiver operating characteristic
AUC	Area-under-the-curve

## References

[1] de Zubiria Salgado A., Herrera-Diaz C. (2012). Lupus nephritis: An overview of recent findings. Autoimmune Dis..

[2] Hanly J.G., O’Keeffe A.G., Su L., Urowitz M.B., Romero-Diaz J., Gordon C., Bae S.C., Bernatsky S., Clarke A.E., Wallace D.J. (2016). The frequency and outcome of lupus nephritis: Results from an international inception cohort study. Rheumatology.

[3] Hoque M., Barman P., Barman S., Roy M., Khan M., Chowdhury M. (2023). A Study on Clinical and Laboratory Status of Active and Inactive LN Patients. Saudi J. Med Pharm. Sci..

[4] Mahajan A., Amelio J., Gairy K., Kaur G., Levy R.A., Roth D., Bass D. (2020). Systemic lupus erythematosus, lupus nephritis and end-stage renal disease: A pragmatic review mapping disease severity and progression. Lupus.

[5] Ayoub I., Cassol C., Almaani S., Rovin B., Parikh S.V. (2019). The kidney biopsy in systemic lupus erythematosus: A view of the past and a vision of the future. Adv. Chronic Kidney Dis..

[6] Petricoin E.F., Zoon K.C., Kohn E.C., Barrett J.C., Liotta L.A. (2002). Clinical proteomics: Translating benchside promise into bedside reality. Nat. Rev. Drug Discov..

[7] Hanash S. (2003). Disease proteomics. Nature.

[8] Rosenblatt K.P., Bryant-Greenwood P., Killian J.K., Mehta A., Geho D., Espina V., Petricoin E.F., Liotta L.A. (2004). Serum proteomics in cancer diagnosis and management. Annu. Rev. Med..

[9] Furey A., Moriarty M., Bane V., Kinsella B., Lehane M. (2013). Ion suppression; a critical review on causes, evaluation, prevention and applications. Talanta.

[10] Ayoglu B., Häggmark A., Neiman M., Igel U., Uhlén M., Schwenk J.M., Nilsson P. (2011). Systematic antibody and antigen-based proteomic profiling with microarrays. Expert Rev. Mol. Diagn..

[11] Patwa T., Li C., Simeone D.M., Lubman D.M. (2010). Glycoprotein analysis using protein microarrays and mass spectrometry. Mass Spectrom. Rev..

[12] Li Q., Zhou J., Wandstrat A., Carr-Johnson F., Branch V., Karp D., Mohan C., Wakeland E., Olsen N. (2007). Protein array autoantibody profiles for insights into systemic lupus erythematosus and incomplete lupus syndromes. Clin. Exp. Immunol..

[13] Haddon D.J., Diep V.K., Price J.V., Limb C., Utz P.J., Balboni I. (2015). Autoantigen microarrays reveal autoantibodies associated with proliferative nephritis and active disease in pediatric systemic lupus erythematosus. Arthritis Res. Ther..

[14] Qi S., Chen Q., Xu D., Xie N., Dai Y. (2018). Clinical application of protein biomarkers in lupus erythematosus and lupus nephritis. Lupus.

[15] Hernández-Neuta I., Neumann F., Brightmeyer J., Ba Tis T., Madaboosi N., Wei Q., Ozcan A., Nilsson M. (2019). Smartphone-based clinical diagnostics: Towards democratization of evidence-based health care. J. Intern. Med..

[16] Guo L., Chen S., Yu Y.L., Wang J.H. (2021). A smartphone optical device for point-of-care testing of glucose and cholesterol using Ag NPs/UiO-66-NH2-based ratiometric fluorescent probe. Anal. Chem..

[17] Priye A., Bird S.W., Light Y.K., Ball C.S., Negrete O.A., Meagher R.J. (2017). A smartphone-based diagnostic platform for rapid detection of Zika, chikungunya, and dengue viruses. Sci. Rep..

[18] Yeo S.J., Choi K., Cuc B.T., Hong N.N., Bao D.T., Ngoc N.M., Le M.Q., Hang N.L.K., Thach N.C., Mallik S.K. (2016). Smartphone-based fluorescent diagnostic system for highly pathogenic H5N1 viruses. Theranostics.

[19] Ludwig S.K., Tokarski C., Lang S.N., van Ginkel L.A., Zhu H., Ozcan A., Nielen M.W. (2015). Calling biomarkers in milk using a protein microarray on your smartphone. PLoS ONE.

[20] Yang G., Li Y., Tang C., Lin F., Wu T., Bao J. (2022). Smartphone-Based Quantitative Analysis of Protein Array Signals for Biomarker Detection in Lupus. Chemosensors.

[21] Hedde P.N., Abram T.J., Jain A., Nakajima R., de Assis R.R., Pearce T., Jasinskas A., Toosky M.N., Khan S., Felgner P.L. (2020). A modular microarray imaging system for highly specific COVID-19 antibody testing. Lab A Chip.

[22] Balbach S., Jiang N., Moreddu R., Dong X., Kurz W., Wang C., Dong J., Yin Y., Butt H., Brischwein M. (2021). Smartphone-based colorimetric detection system for portable health tracking. Anal. Methods.

[23] Demchenko A.P. (2020). Photobleaching of organic fluorophores: Quantitative characterization, mechanisms, protection. Methods Appl. Fluoresc..

[24] Tan G., Baby B., Zhou Y., Wu T. (2022). Emerging molecular markers towards potential diagnostic panels for lupus. Front. Immunol..

[25] Tang C., Zhang S., Teymur A., Yang B., Nazir F., Cai Q., Saxena R., Olsen N.J., Mohan C., Wu T. (2023). V-Set Immunoglobulin Domain–Containing Protein 4 as a Novel Serum Biomarker of Lupus Nephritis and Renal Pathology Activity. Arthritis Rheumatol..

[26] Yuan Y., Qiu J., Lin Z.T., Li W., Haley C., Mui U.N., Ning J., Tyring S.K., Wu T. (2019). Identification of novel autoantibodies associated with psoriatic arthritis. Arthritis Rheumatol..

[27] Otsu N. (1979). A Threshold Selection Method from Gray-Level Histograms. IEEE Trans. Syst. Man. Cybern..

[28] Roy P., Dutta S., Dey N., Dey G., Chakraborty S., Ray R. Adaptive thresholding: A comparative study. Proceedings of the 2014 International Conference on Control, Instrumentation, Communication and Computational Technologies (ICCICCT).

[29] Zar J.H. (1972). Significance testing of the Spearman rank correlation coefficient. J. Am. Stat. Assoc..

[30] Tsokos G.C. (2020). Autoimmunity and organ damage in systemic lupus erythematosus. Nat. Immunol..

[31] Renaudineau Y., Brooks W., Belliere J. (2023). Lupus nephritis risk factors and biomarkers: An update. Int. J. Mol. Sci..

[32] Wolf B.J., Spainhour J.C., Arthur J.M., Janech M.G., Petri M., Oates J.C. (2016). Development of biomarker models to predict outcomes in lupus nephritis. Arthritis Rheumatol..

[33] Zhu H., Luo H., Yan M., Zuo X., Li Q.Z. (2015). Autoantigen microarray for high-throughput autoantibody profiling in systemic lupus erythematosus. Genom. Proteom. Bioinform..

[34] Zhou M., Zhang Y., Liu T., Yang Y., Yang P. Multi-task Learning with Adaptive Global Temporal Structure for Predicting Alzheimer’s Disease Progression. Proceedings of the 31st ACM International Conference on Information & Knowledge Management.

[35] Wang S., Lifson M.A., Inci F., Liang L.G., Sheng Y.F., Demirci U. (2016). Advances in addressing technical challenges of point-of-care diagnostics in resource-limited settings. Expert Rev. Mol. Diagn..

[36] Kolberg J., Penny L., Todd J., Urdea M. (2009). An Assessment of the Technological Issues and Options for Point-of-Care Diagnostic Tests in Resource-Limited Settings.

[37] Ray S., Reddy P.J., Jain R., Gollapalli K., Moiyadi A., Srivastava S. (2011). Proteomic technologies for the identification of disease biomarkers in serum: Advances and challenges ahead. Proteomics.

[38] de Jesus J.R., da Silva Fernandes R., de Souza Pessôa G., Raimundo I.M., Arruda M.A.Z. (2017). Depleting high-abundant and enriching low-abundant proteins in human serum: An evaluation of sample preparation methods using magnetic nanoparticle, chemical depletion and immunoaffinity techniques. Talanta.

[39] Salvi M., Acharya U.R., Molinari F., Meiburger K.M. (2021). The impact of pre-and post-image processing techniques on deep learning frameworks: A comprehensive review for digital pathology image analysis. Comput. Biol. Med..

